# The antivirulence activity, transcriptomics of EGCG and its protective effects on zebrafish infected by *Aeromonas hydrophila*

**DOI:** 10.3389/fcimb.2023.1271448

**Published:** 2023-10-06

**Authors:** Hongmei Yin, Qiaohua Yan, Guoqiang Cheng, Li Zhang, Meiqing Li, Tingting Hu, Sihui Gao, Yuanhang Chen, Huaqiao Tang, Jie Luo

**Affiliations:** ^1^ School of Animal Science, Xichang University, Xichang, Sichuan, China; ^2^ Department of Pharmacy, Sichuan Agricultural University, Chengdu, China; ^3^ Sichuan Academy of Chinese Medicine Sciences, Chengdu, China; ^4^ Key Open Laboratory of Traditional Chinese Veterinary Medicine, Tongren Polytechnic College, Tongren, China

**Keywords:** epigallocatechin-3-gallate, *A. hydrophila*, virulence factors, transcriptome, zebrafish

## Abstract

**Background:**

*Aeromonas hydrophila* is an important pathogen that mainly harms aquatic animals and exhibits resistance to a variety of antibiotics. This study investigated the effect of epigallocatechin-3-gallate (EGCG) on the virulence factors of *A.hydrophila* and its impact on adhesion, invasion, and cytotoxicity in Caco-2 cells. The potential mechanism of antibacterial activity of EGCG was investigated by transcriptomic analysis.

**Results:**

EGCG not only inhibited the production of biofilm, hemolytic activity, motility, and protease activity of *A.hydrophila*, but also reduced its adhesion, invasion, and cytotoxicity in Caco-2 cells. Transcriptomic analysis indicated that the antimicrobial activity of EGCG may be achieved by weakening the chemotaxis and stress response of the bacteria, as well as inhibiting the TonB system. Animal studies demonstrated that EGCG can significantly improve the survival rate and organs damage of zebrafish infected with *A.hydrophila*.

**Conclusion:**

EGCG would be a potential alternative drug for the prevention and treatment of *A. hydrophila* infections by anti-virulence mechanism.

## 1 Introduction

*A.hydrophila* is a common opportunistic gram-negative zoonotic pathogen and widely exists in various aquatic environments. *A.hydrophila* frequently causes hemorrhagic septicemia in farmed and wild fish, manifested by internal and external bleeding, tail rot, exophthalmia, and death after infection (Gao et al., 2019; Pallavi et al., 2021). Human infection with *A.hydrophila* can cause gastroenteritis, hemolytic uremic syndrome, peritonitis, skin infections, meningitis, necrotizing fasciitis, and sepsis in immunocompromised patients (Vilches et al., 2009; Zhu et al., 2020). A variety of virulence factors are related to its pathogenicity, such as adhesins, cytotoxins, proteases, elastases, lipases, hemolysins, lateral and polar flagella, biofilm formation, and exopolysaccharide production (Liu et al., 2019; Li et al., 2020). Preventing the production of virulence factors by pathogens is an important alternative strategy for controlling bacterial infection (Defoirdt, 2018). Antibiotics and chemicals are routinely used to control *A. hydrophila* infections (Chen et al., 2010; Cao et al., 2022). It usually leads to the emergence of “super bacteria”. According to former reports, the resistance rates of aquatic *A.hydrophila* isolated from different sources to β-lactams range from 61.54% to 100%, tetracyclines range from 23.26% to 92.31%, and quinolones range from 6.98% to 100% (Yin et al., 2020). Currently, *A.hydrophila* exhibits a characteristic of broad-spectrum and multidrug resistance to antibiotics (Zhang et al., 2020).

Tea drinking has a long history in China and is an indispensable part of people’s daily lives. Epigallocatechin-3-galate (EGCG), the main active component, accounts for 59% of the total catechins in green tea (Steinmann et al., 2013). EGCG not only has the strongest antibacterial activity but also has anti-inflammatory, anticancer and immunomodulatory effects (Menegazzi et al., 2020). In addition, EGCG exhibits broad-spectrum anti-infective activity against multiple pathogenic microorganisms, but with different mechanisms of action in various bacteria. There are studies reporting that EGCG can inhibit the production of virulence factor by suppressing quorum sensing (QS), thereby reducing the pathogenicity of *Pseudomonas aeruginosa* towards Caenorhabditis elegans (Hao et al., 2021). Tea extract can reduce Fusobacterium nucleatum biofilm formation, hemolysis, and production of virulence factors such as hydrogen sulfide by damaging the cell membrane and chelating iron (Ben Lagha et al., 2017). In Helicobacter pylori, EGCG exerts bactericidal activity through an alternative pathway by interacting with histone-like DNA binding protein, effectively killing the bacteria (Gao et al., 2022).

EGCG has been shown to have a bactericidal effect on multiple pathogenic bacteria, but its effect on *A.hydrophila* has not been studied. We investigated the impact of EGCG on the virulence factors of *A.hydrophila*, including biofilm formation, hemolysis, motility, protease production, and its effect on Caco-2 cells at sub-inhibitory concentrations. Our results demonstrated that EGCG significantly reduced the production of virulence factors and weakened the toxicity of *A.hydrophila* to Caco-2 cells. The transcriptomic analysis revealed that EGCG can directly suppress virulence genes and iron uptake in *A.hydrophila*. Finally, we also studied the protective effect of EGCG on *A.hydrophila* infection in zebrafish, and the results showed that EGCG significantly reduced the pathogenicity of *A.hydrophila.* In conclusion, EGCG is an effective anti-infection agent and a substitute for antibiotics.

## 2 Materials and methods

### 2.1 Bacterial strain, growth condition, and chemicals

The clinical isolate *A.hydrophila AH08* was kindly provided by Prof. Defang Chen (Sichuan Agriculture University) and was used throughout the *in vitro* and *in vivo* studies. The test strain was routinely grown at 30 °C in trypticase soy broth (TSB) with rotary shaking at 150 rpm. All bacterial culture mediums were purchased from Haibo Bio (Qingdao, China). EGCG was obtained from Shanghai Yuanye Bio-Technology Co., Ltd. (Shanghai, China) and dissolved in dimethyl sulfoxide (DMSO) (Biosharp) to prepare stock solutions at concentrations of 80 mg/mL.

### 2.2 Determination of MIC, MBC and growth curves

The MIC and MBC of EGCG were tested against *A.hydrophila* in 96-well plates. Different concentrations of EGCG (ranging from 0 to 256 μg/mL) were incubated with *A.hydrophila* cultures at 30°C with shaking (150 rpm). The OD 600nm value was recorded every hour using a spectrophotometer, and a curve was plotted.

### 2.3 Biofilm inhibition assay

Biofilm formation assays were performed in 96-well plates according to a previous study with slight modification (Yang et al., 2021). Briefly, *A.hydrophila* was cultured in 96-well plates with different concentrations of EGCG (0, 16, 32, 64, 128, and 256 μg/mL) for 24 h or 48 h at 30°C. After incubation, discard the cultures and rinse the samples with PBS three times, taking care to handle them gently. Subsequently, add 200 μL of 0.5% crystal violet for 30 minutes. Following this, dry the plate and dissolve the biofilm stained with crystal violet using 200 μL of 33% acetic acid. The absorbance was measured at 570 nm.

### 2.4 β-hemolysin quantification assay

The extracellular hemolysin production of *A.hydrophila* was quantified according to a previous study with slight modification(Srinivasan et al., 2020). Briefly, 100 µl of both EGCG treated and untreated *A.hydrophila* were incubated with 900 µl of phosphate buffer saline containing 5% rabbit erythrocytes and then incubated for 1 hour at 4°C. The mixture was centrifuged, and then the supernatants were collected and measured at 530 nm.

### 2.5 Motility assays

In the swimming assay, 2 μL overnight cultured *A.hydrophila* was carefully inoculated into swimming agar plates (agar 0.3%, peptone1%, glucose 1%, and NaCl 0.5%) with (256, 128 and 64 μg/mL) or without EGCG added. In the swarming assay, 6 μL of bacterial suspension was pipetted onto the center of the prepared swarming agar plates (agar 0.5%, peptone 1%, NaCl 0.5%, and glucose 0.5%) in the presence or absence of EGCG. The migrated zones were recorded after 18 h of incubation at 30 °C.

### 2.6 Proteinase activity

The effect of EGCG on the proteolytic activity of *A.hydrophila* was evaluated using the skim milk agar plate method. Skim milk agar plates containing different concentrations of EGCG and 2% skim milk were prepared. Overnight bacterial cultures were diluted to 1×10^9^ CFU/mL and 20 µL were inoculated onto the center of the plates. The plates were incubated at 30°C for 24 hours. Protease activity was quantified by calculating the ratio of the diameter of the clearance zone to the diameter of the bacterial colony.

### 2.7 Scanning electron microscopy

Cut medical tubing into 1 cm lengths and place them in a 6-well microtiter plate. Mix the tubing with bacterial cultures and different concentrations of EGCG (256, 128, and 64 μg/mL), and incubate for 24 hours. Subsequently, the tubing was washed multiple times with PBS, immersed in 2.5% glutaraldehyde for at least 24 hours, and then dried and dehydrated with 30%, 50%, 70%, 90% and 100% ethanol. The finished tubing was sprayed with gold and observed under scanning electron microscope (Tescan Mira4, China).

### 2.8 Cell experiment

Caco-2 cell was obtained from Shanghai Zhongqiaoxinzhou Biotechnology Co.,Ltd.(Shanghai, China). Cells were cultured in DMEM (Basalmedia, Shanghai, China) supplemented with 20% (v/v) fetal bovine serum (FBS, NEWZERUM, China) and 5% antibiotics (penicillin and streptomycin, Basalmedia, Shanghai, China). Caco-2 cells were seeded at a concentration of 5 × 10^4^ cells per well in 24-well or 96-well cell culture plates to obtain a monolayer of differentiated cells.

The adhesion and invasion experiments refer to the previous studies with minor modifications (Schierack et al., 2011). After incubating *A.hydrophila* with EGCG for 24 hours, the mixture was centrifuged and resuspended in DMEM to adjust the bacterial concentration to 1×10^8^ CFU/mL. The bacterial suspension was then added to a monolayer of differentiated Caco-2 cells and incubated for 1 hour. In the adhesion experiments, the culture medium was discarded, and the cells were washed three times with PBS (pH 7.4) before adding 0.25% trypsin for digestion. After gradient dilution, bacterial counting was performed. In the invasion experiments, the culture medium was discarded, and 10% antibiotics (penicillin and streptomycin) were added for 1 hour before bacterial counting was performed. LDH release was tested according to the manufacturer’s instructions (Nanjing Jiancheng Bioengineering Institute, Jiangsu, China).

### 2.9 RNA isolation and sequencing

*A.hydrophila* was cultured for 12 h with or without EGCG (256µg/mL), and total RNA was extracted from the culture using TRIzol reagent (TransGen Biotech, Beijing, China). Agarose gel electrophoresis and Bioanalyzer 2100 system (Agilent Technologies, CA, USA) were used to examine whether the RNA had been contaminated or degraded. Following fragmentation, the cDNA library was constructed and sequenced on an Illumina Novaseq platform at the Novogene Bioinformatics Institute (Beijing, China).

### 2.10 RNA-Seq data processing and bioinformatics

Sequence information and quality were evaluated through the establishment of a library and the original sequencing obtained by high-throughput sequencing. To ensure that analysis requirements were met, low quality spliced raw reads were filtered to obtain high quality clean reads for subsequent sequencing analysis. The clean reads were then aligned to the reference genome (GCF_022982835.1) of *A. hydrophila* using bowtie2 (ver. 2.3.4.3). HTSeq v0.6.1 was used to count the reads numbers mapped to each gene. And then FPKM of each gene was calculated based on the length of the gene and reads count mapped to this gene. Differential expression analysis was performed using R package DESeq2 (ver. 1.20). Genes with an adjusted p-value of <0.05 found by DESeq were assigned as differentially expressed. Gene ontology (GO) enrichment analysis and Kyoto Encyclopedia of Genes and Genomes (KEGG) pathway analysis of differentially expressed genes were implemented by the R package cluster profile (ver. 3.8.1).

### 2.11 qRT-PCR

Total RNA was extracted from the EGCG-treated *A.hydrophila* cultures by using TransZol Up (TransGen Biotech, Beijing, China), and cDNA synthesis was conducted by using a reverse transcription kit (ThermoFisher Scientific, Waltham, MA, USA). UV spectrophotometer (Implen, Munich, Germany) is used to determine the concentration and purity of RNA. qRT−PCR was performed on a LightCycler® 480II Master (Roche, Germany) with a final reaction volume of 10 μL. The primer pairs used in this study are shown in **Table S1** (*A.hydrophila*) and **Table S2** (zebrafish).

### 2.12 Animal experiment

All animal experiments were conducted under the guidance of the Animal Welfare and Ethics Committee of Sichuan Agricultural University (Approval No. 20230045). Clinically healthy wild-type adult zebrafish were collected from Chongqing Fish Aquarium. All zebrafish were acclimatized at 28 ± 2 °C for 5 days in a sterilized glass aquarium and fed commercial food pellets twice a day. Zebrafish were randomly divided into five groups (n=18): vehicle, bacteria only, and bacteria + EGCG (10 mg/kg, 50 mg/kg and 100 mg/kg). *A.hydrophila* was incubated in nutrient broth to exponential phase, washed with sterile PBS buffer three times and resuspended in PBS to 5 × 10^8^ CFU/mL. Bacteria (10 μl, 5 × 10^8^ CFU/mL) were injected into the abdominal cavity of zebrafish, then EGCG was injected, and the control group was injected with the same amount of normal saline. Record clinical symptoms and mortality rate every 6 hours after bacterial infection. Six hours after bacterial challenge, repeat the random selection of 4 zebrafish for bacterial load assessment and histopathological examination. And rapidly collect intestinal tissue from zebrafish and use qRT-PCR to detect gene expression of IL-1β, IL-8, IL-6 and TNF-α.

### 2.13 Statistical analysis

The *in vitro* assays were performed in triplicate, and all data are presented as the mean ± SEM. Statistical analyses were performed by one-way ANVOA posted with Tukey’s multiple comparisons (IBM SPSS statistics 25, IBM, USA). The following terminology is used to denote statistical significance: *p <0.05; **p <0.01.

## 3 Results

### 3.1 EGCG inhibits the virulence of *A.hydrophila*

**Figure 1:**
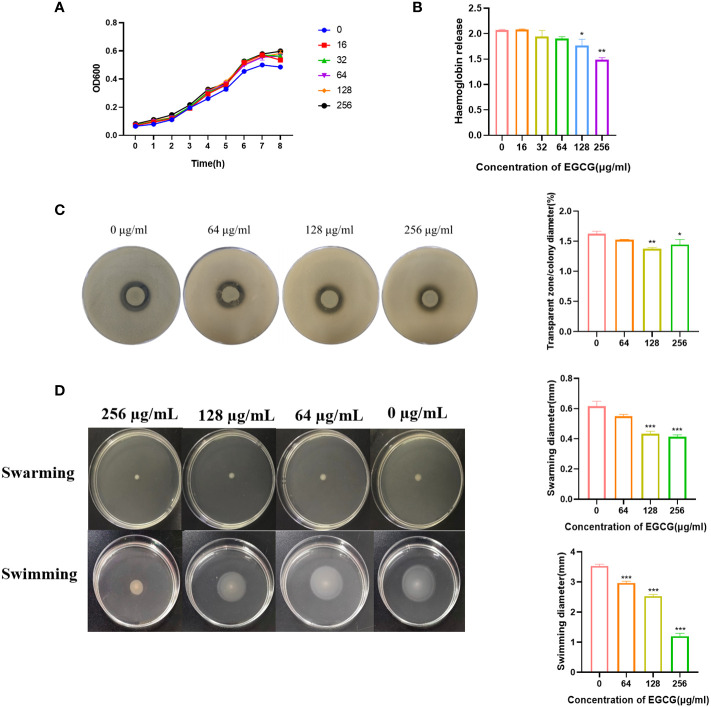
**(A)** Growth curve of *A. hydrophila*. **(B)** Hemolysis activity. **(C)** Inhibitory effect of EGCG on proteolytic enzymes of *A.hydrophila*. **(D)** Effect of EGCG on swarming and swimming motility of *A. hydrophila*. All data are representative of three independent experiments performed in triplicate and expressed as the mean ± SEM values in each bar. * p < 0.05; ** p < 0.01; *** p < 0.001.

The MIC and MBC of EGCG against *A.hydrophila* were 1024 μg/mL and 4096 μg/mL, respectively. All the following experiments were conducted at sub-MICs (256, 128, 64, 32 and 16 μg/mL). The growth curve showed that the addition of EGCG had no effect on the growth of *A.hydrophila* (**Figure 1A**).

Hemolysin is the main virulence factor of *A.hydrophila*. As shown in **Figure 1B**, a high concentration of EGCG could significantly inhibit the hemolytic activity of *A.hydrophila*. Compared with the blank, the inhibition rate of 256 μg/mL EGCG on hemolysin was 28.2%.

The inhibitory effect of EGCG on the production of protease in *A.hydrophila* was evaluated using the skim milk agar plate method. As the concentration of EGCG increased, the diameter of the transparent zone gradually decreased, and the boundary became blurred, but the colony diameter showed no significant change (**Figure 1C**). High concentrations of EGCG significantly inhibited the production of protease in *A.hydrophila.*

EGCG significantly inhibited the motility of *A.hydrophila* in a concentration dependent manner (**Figure 1D**). In swarming, EGCG at 128 µg/mL and 256 µg/mL inhibited *A. hydrophila* by 29.72% and 32.97%, respectively. The inhibition rates of EGCG in swimming, 64 µg/mL, 128 µg/mL and 256 µg/mL were 16.03%, 28.30% and 66.03%, respectively.

**Figure 2:**
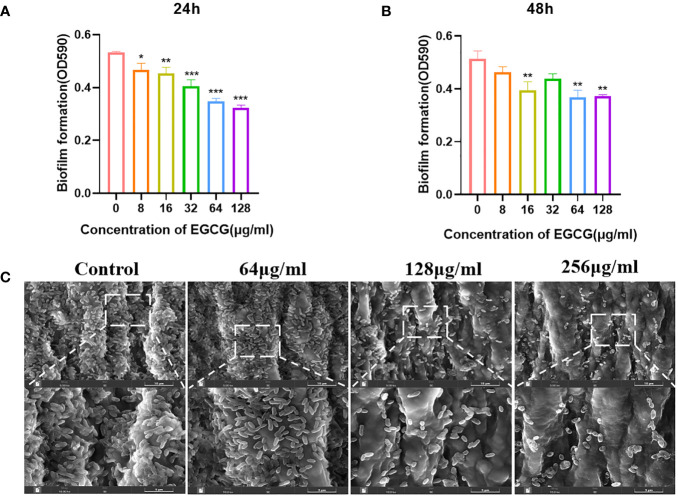
**(A, B)** Effect of 24 h and 48 h EGCG on biofilm formation. **(C)** SEM of biofilm. All data are representative of three independent experiments performed in triplicate and expressed as the mean ± SEM values in each bar. * p < 0.05; ** p < 0.01; *** p < 0.001.

As shown in **Figures 2A, B**, EGCG significantly inhibited the formation of *A.hydrophila* biofilms. When EGCG was 128 μg/mL, the inhibition rate of biofilm formation was 38.27% at 24 h and 26.62% at 48 h. At 24 h, EGCG inhibited biofilm formation in a concentration dependent manner.

SEM provides a visual representation of the growth state of biofilms. The control group biofilm was dense and adhered to the entire medical tubing (**Figure 2C**). After treatment with EGCG, the biofilm was significantly reduced, and the bacteria were scattered in the attachment. This result is consistent with the crystal violet experiment.

### 3.2 EGCG reduces the toxicity of *A.hydrophila* to Caco-2

**Figure 3:**
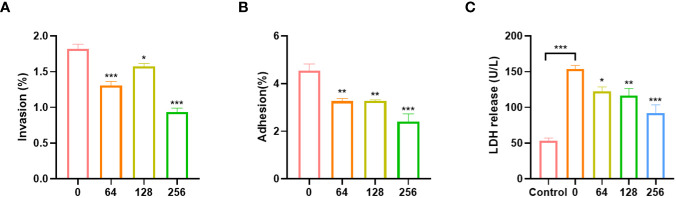
Effects of *A.hydrophila* incubated with EGCG on **(A)** invasion, **(B)** adhesion efficiency and **(C)** LDH release of Caco-2. All data are representative of three independent experiments performed in triplicate and expressed as the mean ± SEM values in each bar. * p < 0.05; ** p < 0.01; *** p < 0.001.

EGCG can inhibit the adhesion and invasion of *A.hydrophila* to Caco-2 cells. The adhesion and invasion rates of untreated *A.hydrophila* to Caco-2 cells were 4.54% and 1.82%, respectively (**Figures 3A, B**). After treatment with EGCG, the adhesion and invasion abilities of *A.hydrophila* to Caco-2 cells were significantly reduced. At a concentration of 256 µg/mL of EGCG, the adhesion and invasion rates were 2.40% and 0.93%, respectively, which was a two-fold reduction compared to the control group. The cytotoxicity of *A.hydrophila* on Caco-2 cells was assessed by measuring the LDH release from infected cells (**Figure 3C**). As compared to the PBS-treated group, *A.hydrophila* treatment resulted in a significant increase in LDH release, indicating its ability to disrupt cellular integrity and cause toxicity in Caco-2 cells. Treatment with EGCG led to a gradual decrease in LDH release, with a concentration-dependent effect. At a concentration of 256 µg/mL, EGCG reduced LDH release by 40%.

### 3.3 Transcriptome changes in *A. hydrophila* after EGCG treatment

**Figure 4:**
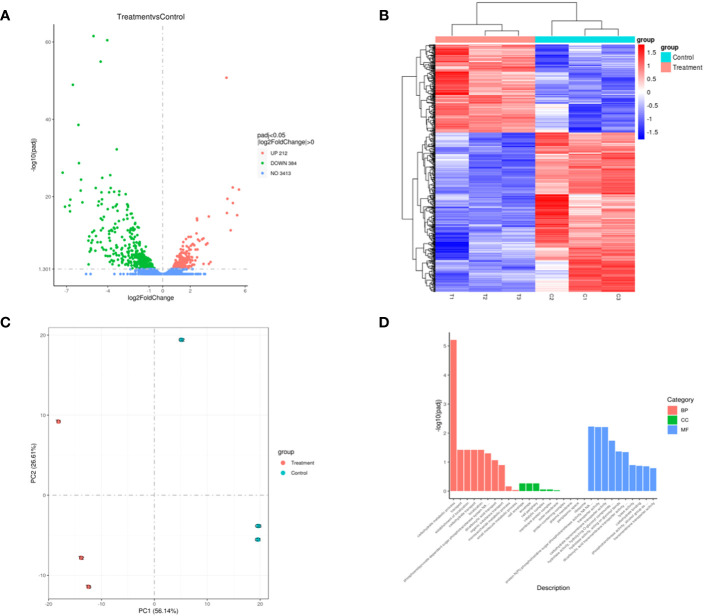
Transcriptome changes in *A. hydrophila* after EGCG treatment. **(A)** Volcano map of differentially expressed genes; **(B)** heatmap of the differentially expressed genes in *A. hydrophila* treated or untreated with EGCG; **(C)** PCA plot of differentially expressed gene. **(D)** Bar diagram of GO enrichment.

The transcriptome sequencing was performed on the RNA of the control group (C) and the 256 µg/mL EGCG-treated group (T). We obtained about 7.1 G of clean data by transcriptome sequencing, including 47,069,472 rawreads and 46,225,720 clean reads (**Table S3**). The error rate of single base location sequencing in all six groups was less than 1%. Transcriptome analysis identified 596 differentially expressed genes between umbelliferone treated and untreated *A. hydrophila*, of which upregulated and downregulated genes were 212 and 384, respectively (**Figure 4A**; **Table S4**). The results of hierarchical cluster analysis (**Figure 4B**) and principal component analysis (PCA) (**Figure 4C**) showed that six different samples were clustered into two groups according to DEGs expression data. Compared with the control group, treatment with EGCG resulted in significant downregulation of genes related to the virulence, stress response, and TonB system of *A.hydrophila* (**Table 1**). Conversely, genes related to purine metabolism, amino acid metabolism, and energy metabolism were upregulated (**Table 2**). GO analysis of differentially expressed genes showed that the downregulated genes were mainly involved in metabolism, transport, and localization, while the upregulated genes were mainly involved in signal transduction (**Figure 4D**).

**Table 1:** The representative downregulated gene list.

	Gene Name	Log2 Fold Change	Description
**Genes associated with bacterial virulence**	MUW98_RS02610	-2.462476078	SGNH/GDSL hydrolase family protein
	MUW98_RS20835	-1.537520536	triacylglycerol lipase
	MUW98_RS04750	-2.189222336	methyl-accepting chemotaxis protein
	MUW98_RS09395	-1.877322408	methyl-accepting chemotaxis protein
	MUW98_RS19490	-1.749066889	methyl-accepting chemotaxis protein
	MUW98_RS11305	-1.427660893	methyl-accepting chemotaxis protein
	MUW98_RS20975	-1.17387116	HlyD family efflux transporter periplasmic adaptor subunit
	flgL	-1.331271215	flagellar hook-associated protein FlgL
	MUW98_RS09510	-1.379102273	HBL/NHE enterotoxin family protein
	ompA	-2.113016824	OmpA family
**Genes associated with bacterial stress**	MUW98_RS00255	-2.120122177	universal stress protein
	MUW98_RS04890	-2.549077628	universal stress protein
	uspE	-1.209510175	universal stress protein UspE
**Genes associated with the TonB system**	MUW98_RS10140	-6.099936787	siderophore amonabactin TonB-dependent receptor
	MUW98_RS18490	-5.130488102	TonB-dependent hemoglobin/transferrin/lactoferrin family receptor
	MUW98_RS23235	-4.450289001	TonB-dependent siderophore receptor
	MUW98_RS23100	-3.900517826	energy transducer TonB
	MUW98_RS13085	-1.89207427	TonB-dependent siderophore receptor
	MUW98_RS01650	-3.772549715	ligand-gated channel protein
	MUW98_RS04825	-1.290015165	TonB-dependent receptor
	MUW98_RS23110	-3.311906893	MotA/TolQ/ExbB proton channel family protein
	MUW98_RS10215	-3.195409569	biopolymer transporter ExbD
	MUW98_RS23115	-4.461527197	MotA/TolQ/ExbB proton channel family protein
	MUW98_RS23105	-4.361256322	biopolymer transporter ExbD

**Table 2:** The representative upregulated gene list.

	Gene Name	Log2 Fold Change	Description
**Genes involved in purine metabolism**	guaD	4.709089932	guanine deaminase
	MUW98_RS11050	4.641481341	purine permease
	xdhC	3.482191635	xanthine dehydrogenase iron sulfur-binding subunit XdhC
	xdhA	1.950715135	xanthine dehydrogenase molybdenum-binding subunit XdhA
	xdhB	2.676050177	xanthine dehydrogenase FAD-binding subunit XdhB
**Genes related to amino acid biosynthesis**	arcC	5.108238112	carbamate kinase
	ygfM	5.096347305	molybdopterin-dependent oxidoreductase FAD-binding subunit
	argB	2.287231102	acetylglutamate kinase
	ssnA	5.417331398	putative aminohydrolase SsnA
	MUW98_RS20405	1.871977483	argininosuccinate synthase
**Genes related to energy metabolism**	MUW98_RS18685	2.037008822	fatty acid CoA ligase family protein
	acs	1.949341332	acetate–CoA ligase
	MUW98_RS11100	3.151778092	acyl-CoA synthetase

### 3.4 qRT-PCR

**Figure 5:**
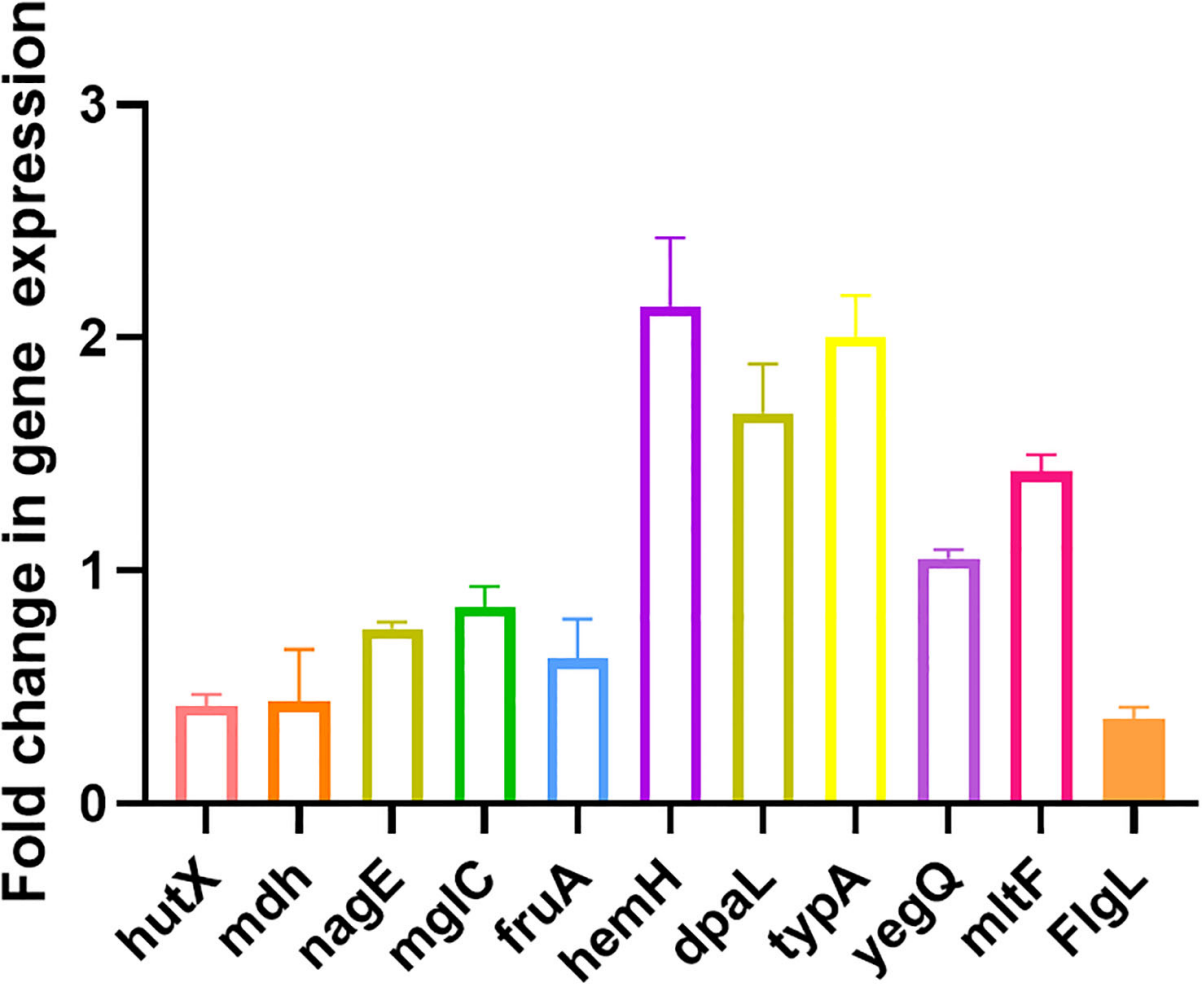
Validation of expression levels of 10 randomly selected genes by qRT-PCR compared with the transcriptome results. All data are representative of three independent experiments performed in triplicate and expressed as the mean ± SEM values in each bar.

Ten randomly selected genes were validated for their expression using qRT-PCR method to confirm the reliability of transcriptome data. All selected genes showed consistent results between RNA sequencing and qRT-PCR data (**Figure 5**).

### 3.5 Protective effect of EGCG against *A.hydrophila* infection in zebrafish

**Figure 6:**
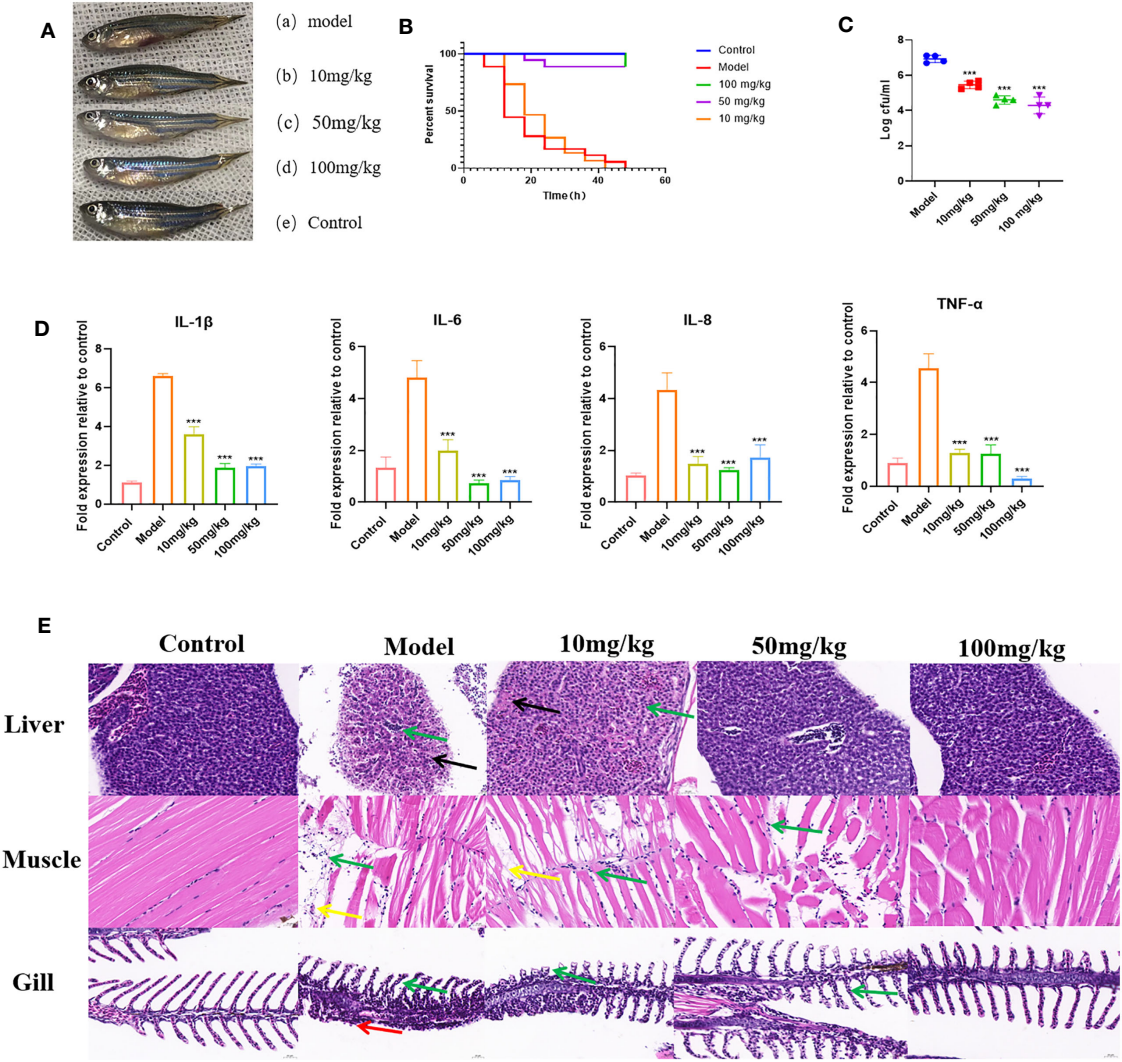
Protective effect of EGCG on zebrafish infected with *A. hydrophila*. **(A)** EGCG alleviates the symptoms of zebrafish infected with *A.hydrophila*. **(B)** EGCG increases the survival rate of zebrafish infected with *A.hydrophila* (n=18). **(C)** The bacterial load of zebrafish treated with EGCG decreased significantly (n=4). **(D)** EGCG significantly reduced the production of intestinal IL-1β, IL-6, IL-8 and TNF-α cytokines in zebrafish (n=6). **(E)** Histopathological analysis of selected tissues from various groups. The sections were stained with H&E. Inflammatory cell infiltration (green arrow), necrosis of liver cells (black arrows), muscle fiber rupture (yellow arrow), necrosis of the lamellar epithelial cells of the gill filaments (red arrow). All data are representative of three independent experiments performed in triplicate and expressed as the mean ± SD values in each bar. * p < 0.05; ** p < 0.01; *** p < 0.001.

To further demonstrate the inhibitory effect of EGCG on the virulence of *A.hydrophila*, we conducted *in vivo* experiments using a zebrafish infection model. The effect of EGCG on the survival of zebrafish challenged with *A.hydrophila* was assessed. The uninfected control group showed 100% survival at 48 h. Zebrafish began to die 6 h after intraperitoneal injection of *A.hydrophila*. The dead zebrafish showed abdominal bleeding, ulceration, and edema, and some zebrafish showed eye bleeding and other symptoms (**Figure 6A**). At 48 h after infection, the survival rate of zebrafish in the model group was 0, while that in the high-dose and medium-dose groups was as high as 88.9%. In contrast, the low-dose group showed the same survival rate as the model group (**Figure 6B**).

The surface adherent cells of *A. hydrophila* were recovered from post-challenged zebrafish to assess the influence of EGCG in the treatment groups (**Figure 6C**). The results showed that the adhesion of *A.hydrophila* to zebrafish was significantly reduced after EGCG treatment. Compared with the model group, the 10 mg/kg, 50 mg/kg and 100 mg/kg EGCG bacterial adhesion CFU were decreased by 21.22%, 33.47% and 38.00%, respectively. Although the low concentration of EGCG did not improve the survival rate of zebrafish, it still reduced the bacterial adhesion rate.

To investigate whether EGCG plays an immunomodulatory role, we detected the production of *IL-1β*, *IL-6*, *IL-8* and *TNF-α* in the intestinal tissue of zebrafish. As shown, 6 h after intraperitoneal injection of *A.hydrophila*, zebrafish intestinal tissue showed a cytokine surge. However, EGCG treatment significantly inhibited the expression of the proinflammatory cytokine genes *IL-1β*, *IL-6*, *IL-8* and *TNF-α* (**Figure 6D**). The results showed that EGCG could inhibit the strong inflammatory response caused by bacterial infection.

Histopathological examination showed that *A.hydrophila* infection resulted in significant pathological changes. In the model group, a large number of inflammatory cells infiltrated all tissues, indicating that *A.hydrophila* infection leads to a systemic inflammatory response. EGCG conferred significant protection against the side effects on the tissues of *A.hydrophila* infected zebrafish in a dose-dependent manner (**Figure 6E**).

## 4 Discussion

The pathogenicity of *A.hydrophila* is closely associated with its virulence factors. The biofilm is an organized microbial aggregate that helps microbes resist extreme environments and is the cause of many persistent and chronic infections. The initial step in biofilm formation is the reversible attachment of planktonic bacteria to a surface, followed by irreversible attachment and proliferation to form microcolonies that produce extracellular polymeric substance, resulting in the mature biofilm. Biofilm is one of the targets to reduce bacterial resistance. The formation of biofilms is considered to be associated with the motility of bacteria. In Escherichia coli, flagellar activity has been proposed to facilitate initial cell-surface contact, which may help to overcome repulsive forces on the surface. The locomotion of bacteria can also enable bacteria to move out of adverse environments, and this swimming is associated with rotating polar flagella and transverse flagella. Our results demonstrate that EGCG significantly inhibits biofilm formation and motility of *A.hydrophila.* Hemolysin and protease are critical extracellular virulence factors produced by *A. hydrophila*, which can disrupt host tissue, allowing the pathogen to obtain nutrients and disseminate. In our study, high concentrations of EGCG significantly inhibited the hemolytic and proteolytic activities of *A. hydrophila*.

After ingestion of food contaminated with *A.hydrophila*, humans may develop symptoms including watery or bloody diarrhea, subacute or chronic gastroenteritis, and cholera-like illness. Caco-2 cells can spontaneously differentiate and form a mature monolayer of intestinal cells in culture medium, which is commonly used as an *in vitro* model to simulate the intestinal barrier. This study employed monolayer differentiated intestinal epithelial cells to evaluate the effects of EGCG on adherence, invasion, and cytotoxicity of *A.hydrophila.* Adherence of pathogens to the host surfaces and tissues is an essential initial step for most infections. After adherence to the intestinal epithelium, bacteria can invade and subsequently translocate across the mucosal epithelial cells, forming physical barriers of the cell membrane and intercalating tight junctions. Inhibition of bacterial adhesion may be an effective strategy for preventing infection. Our results indicate that co-cultivation of *A.hydrophila* with EGCG significantly reduced its adhesion and invasion rates on Caco-2 cells. After adhering to intestinal epithelial cells, *A.hydrophila* produces numerous potential virulence factors to disrupt the epithelial barrier and impair immune cells. The LDH assay results indicate that *A.hydrophila* can compromise the integrity of Caco-2 cell membrane, while such damage is significantly reduced upon treatment with EGCG.

To further investigate the inhibitory mechanism of EGCG on *A.hydrophila* virulence, we compared the gene expression profile with or without EGCG treatment using transcriptomic analysis. We found that EGCG treatment significantly downregulated a series of virulence factors, stress proteins, and TonB system-related genes. Firstly, genes directly related to the pathogenicity of *A.hydrophila*, including lipase, enterotoxin, flagella, and OmpA genes, were downregulated by EGCG. Secondly, the gens for the expression of multiple methyl-accepting chemotaxis proteins (MCPs) were also inhibited by EGCG. MCPs are involved in regulating various aspects of cell activity, including biofilm formation, flagellar biosynthesis, extracellular polysaccharide production, and toxin production. Additionally, EGCG downregulates the expression of stress proteins. Stress proteins aid bacteria in surviving under stress conditions and participate in cell growth and survival, motility, biofilm formation, and pathogenicity towards hosts. Iron is an essential trace nutrient required for bacterial survival within host tissues, and it participates in the expression of virulence factors, such as biofilm formation, motility, and invasion. Active transport of iron in Gram-negative bacteria is mediated by the TonB complex (TonB-ExbB-ExbD) and TonB-dependent transporters. Additionally, TonB-mediated active transport of nutrients is crucial for the survival of pathogenic bacteria during infection. Studies have shown that in *A.hydrophila*, inactivation of TonB123 affects the motility, adhesion, biofilm formation, and invasiveness of the bacterium. Our results indicate that TonB-related receptors and genes associated with the ExbB-ExbD proton channel were downregulated, suggesting that the drug can affect iron uptake. The effects of EGCG on *A.hydrophila* are multifaceted, including the direct downregulation of virulence-related genes, the weakening of bacterial stress responses, and the downregulation of the TonB system. Additionally, EGCG also affects carbon metabolism, sugar metabolism, and other cellular processes.

To further evaluated the anti-infection effect, an *in vivo* experiment was conducted on an *A.hydrophila* infection model of zebrafish. The results of the *in vivo* experiment showed that EGCG significantly increased the survival rate of zebrafish and reduced the infection rate of *A.hydrophila* compared with the model group. In addition, compared with the untreated control, EGCG treatment decreased the *A.hydrophila* colonization. After *A.hydrophila* infection, the abdomen of zebrafish exhibited ulceration and bleeding, and the amount of proinflammatory cytokines in the model group increased significantly. According to the qRT−PCR results, EGCG inhibited the transcription of the proinflammatory cytokine genes TNF-α, IL-1β, IL-8 and IL-6 of zebrafish intestine. These results indicated that EGCG obtained strong ability to inhibit the overproduction of proinflammatory cytokines and overactivation of the zebrafish innate immune system, thereby significantly reducing mortality from severe bacterial infections. In line with this result, histopathological sections showed that EGCG significantly reduced inflammatory cell infiltration in the liver, muscle, and gill of zebrafish. Administration of EGCG obviously improved the liver cord disorder, vacuolar degeneration or necrosis, muscle fibers break and dissolve, gill tissue structure destroy, and the epithelial cells necrotic that caused by *A. hydrophila*.

## 5 Conclusion

The sub-MICs of EGCG could significantly inhibit the virulence of *A. hydrophila in vitro*, increase the survival rate of zebrafish infected with *A. hydrophila*, and effectively inhibited the bacterial colonization and inflammation. The transcriptomic results suggest that the potential pathways mediating virulence inhibition of EGCG may relate to the attenuation of bacterial chemotaxis and stress response, as well as the inhibition of the TonB system. Therefore, EGCG may be a potential alternative drug for the prevention and treatment of *A. hydrophila* infections.

## Data availability statement

The datasets presented in this study can be found in online repositories. The names of the repository/repositories and accession number(s) can be found in the article/**Supplementary Material**.

## Ethics statement

The animal study was approved by the Institutional Animal Care and Use Committee of Sichuan Agricultural University. The study was conducted in accordance with the local legislation and institutional requirements.

## Author contributions

QY: Data curation, Formal Analysis, Methodology, Writing – original draft. HY: Formal Analysis, Funding acquisition, Methodology, Writing – original draft. GC: Methodology, Writing – original draft. LZ: Software, Writing – original draft. ML: Validation, Writing – original draft. TH: Validation, Writing – original draft. SG: Validation, Writing – original draft. YC: Validation, Writing – original draft. HT: Conceptualization, Data curation, Supervision, Writing – review & editing. JL: Conceptualization, Funding acquisition, Writing – review & editing.
