# Supplementary material for: The antivirulence activity, transcriptomics of EGCG and its protective effects on zebrafish infected by Aeromonas hydrophila
Source: Front Cell Infect Microbiol. 2023 Oct 6;13:1271448. doi: 10.3389/fcimb.2023.1271448 (PMC10587681; doi:10.3389/fcimb.2023.1271448)
Supplement: Supplementary file 1 [file Table_1.docx]

Table S1 The primers for zebrafish proinflammatory cytokines

| Name | Forward (5′-3′) | Reverse (3′-5′) |
| --- | --- | --- |
| IL-1β | TGGACTTCGCAGCACAAAATG | GTTCACTTCACGCTCTTGGATG |
| IL-8 | GTCGCTGCATTGAAACAGAA | CTTAACCCATGGAGCAGAGG |
| IL-6 | ACGACATCAAACACAGCACC | TCGATCATCACGCTGGAGAA |
| TNF-α | GCTGGATCTTCAAAGTCGGGTGTA | TGTGAGTCTCAGCACACTTCCATC |
| β-actin | CCATCTATGAGGGTTACGC | GACAATTTCTCTTTCGGCT |

Table S2 The primers for Aeromonas hydrophila

| Name | Forward (5′-3′) | Reverse (3′-5′) |
| --- | --- | --- |
| arcC | AATGCTCTGCTCCGTCGTGG | GATACCTTGGTGTAGGCGCTGTT |
| mdh | CGGCATCATCACCAACCC | CGAAGGTCTCGGCACGGA |
| dpaL | TGCCCCTGTTTTCCCGT | GTTGGCGATGGCGTAGACCC |
| typA | CCTGCGTAAGGGCGAGATGA | GTGCGGACCGTAGTGG |
| mglC | TGGTCTGGTCAACGGTCTCAT | GTTCTTGCCAAAGCGGGTCT |
| nagE | CGTAACCTCCGTGGTGATGA | AAGTTCGGGATGTCGTTGATG |
| yegQ | GCTGGTGCTGCTGGAAGAGTC | GCCCATCCTGGTCAGTCGTT |
| mltF | CAAGGTACGCTACGGCTATGC | GCTCGCTGCTGTCGTCATC |
| hemH | TGTCCTCTGCTCCACTTCATCATC | GCTGCTGCTTGCTTATCACCATC |
| 16S rRNA | CAACCCCTGTCCTTTGTT | TTTGGGATTCGCTCACTA |

Table S3 Summary of RNA-seq alignment.

| Sample | Total reads | Clean reads | Error rate% | Q20（100%） | Q30（100%） | GC% |
| --- | --- | --- | --- | --- | --- | --- |
| T1 | 7851654 | 7718876 | 0.03 | 97.26 | 92.57 | 58.37 |
| T2 | 7596326 | 7426120 | 0.03 | 97.22 | 92.51 | 59.16 |
| T3 | 7970074 | 7842446 | 0.03 | 96.98 | 91.98 | 59.69 |
| C1 | 7806874 | 7617012 | 0.03 | 97.11 | 92.28 | 59.62 |
| C2 | 7847218 | 7728776 | 0.03 | 97.43 | 92.85 | 57.68 |
| C3 | 7997326 | 7892490 | 0.03 | 97.18 | 92.4 | 59.6 |

Q20: percentage of bases with a Phred value > 20; Q30: percentage of bases with a Phred value > 30.
